# Higher Pathological Complete Response Rate of Less than 10 Total Axillary Lymph Nodes After Axillary Lymph Node Dissection Following Neoadjuvant Chemotherapy in Breast Cancer

**DOI:** 10.3389/fsurg.2022.678169

**Published:** 2022-03-31

**Authors:** Jeeyeon Lee, Nora Jee-Young Park, Byeongju Kang, Jin Hyang Jung, Wan Wook Kim, Yee Soo Chae, Soo Jung Lee, Hye Jung Kim, Ji-Young Park, Ho Yong Park

**Affiliations:** ^1^Department of Surgery, School of Medicine, Kyungpook National University, Daegu, South Korea; ^2^Kyungpook National University Chilgok Hospital, Daegu, South Korea; ^3^Department of Pathology, School of Medicine, Kyungpook National University, Daegu, South Korea; ^4^Department of Oncology/Hematology, School of Medicine, Kyungpook National University, Daegu, South Korea; ^5^Department of Radiology, School of Medicine, Kyungpook National University, Daegu, South Korea

**Keywords:** breast cancer, axillary lymph node, neoadjuvant chemotherapy, pathological complete response, dissection

## Abstract

**Background:**

The American Joint Committee on Cancer (AJCC) guideline recommends the evaluation of ≥10 axillary lymph nodes (ALN) in patients with breast cancer to assess the N stage. However, the total ALN count in ALN dissection (ALND) often decreases after neoadjuvant chemotherapy in breast cancer. The authors compared clinicopathological factors and oncological outcomes between <10 vs. ≥10 ALNs after ALND following neoadjuvant chemotherapy in breast cancer.

**Methods:**

Data of 159 patients with breast cancer, treated with neoadjuvant chemotherapy and ALND, were reviewed, and the cases were classified into two groups (<10 vs. ≥10 ALN count). The treatment response was determined based on the RECIST 1.1 criteria, and histopathological regression of the tumor was assessed based on the Miller-Payne grading scales.

**Results:**

Most of the clinical and pathological factors did not demonstrate any significant differences between the two groups. However, the pathological complete response (pCR) rate in breast lesion and ALNs were the higher trend in the group with <10 ALNs. During the 88-month follow-up period, there was no significant difference in locoregional recurrence, distant metastasis, or overall survival.

**Conclusions:**

Although there was a limitation due to different sample sizes, additional axillary surgery may not be necessary even in cases with <10 total ALNs after ALND, following neoadjuvant chemotherapy because the lymph nodes are more likely to have been regressed themselves due to neoadjuvant chemotherapy, and the residual lymph nodes may be absent.

## Background

Recent advanced treatment strategies developed for breast cancer have improved the prognosis of patients with breast cancer. Based on the National Surgical Adjuvant Breast and Bowel Project B-18 and B-27 results, neoadjuvant chemotherapy (NAC) is considered before surgery in locally advanced breast cancer, which has allowed not only better oncological outcomes, but also a high rate of breast conservation in surgery (1, 2). However, it is important to perceive the clinical stage and histological characteristics before initiation of treatment, because those may change, owing to NAC.

The goal of NAC in breast cancer is pathological complete response (pCR) for breast or axillary metastatic lesions. When the pCR is achieved in the breast or axillary lesions, the prognosis of breast cancer becomes better (1, 3–5). However, the normal structures are also damaged along with the breast cancer during NAC. The edematous breast parenchyma or fibrotic change in the axillary area is a common surgical finding in breast cancer managed with NAC. In addition, normal lymph node structures are usually denatured, and, as a result, the total lymph node count may decrease.

Based on the AJCC staging guideline, at least 10 total lymph nodes should be evaluated for accurate determination of the N stage (6–8). However, occasionally, the total lymph node count is reported as <10 after NAC in breast cancer, even if the complete axillary lymph node dissection (ALND) was performed by a well-experienced surgeon.

We compared the oncological outcomes of breast cancer treated with NAC between groups with <10 and 10 or more total lymph nodes after ALND, identifying pathological results and evaluating the associated clinical factors.

## Methods

**Figure 1:**
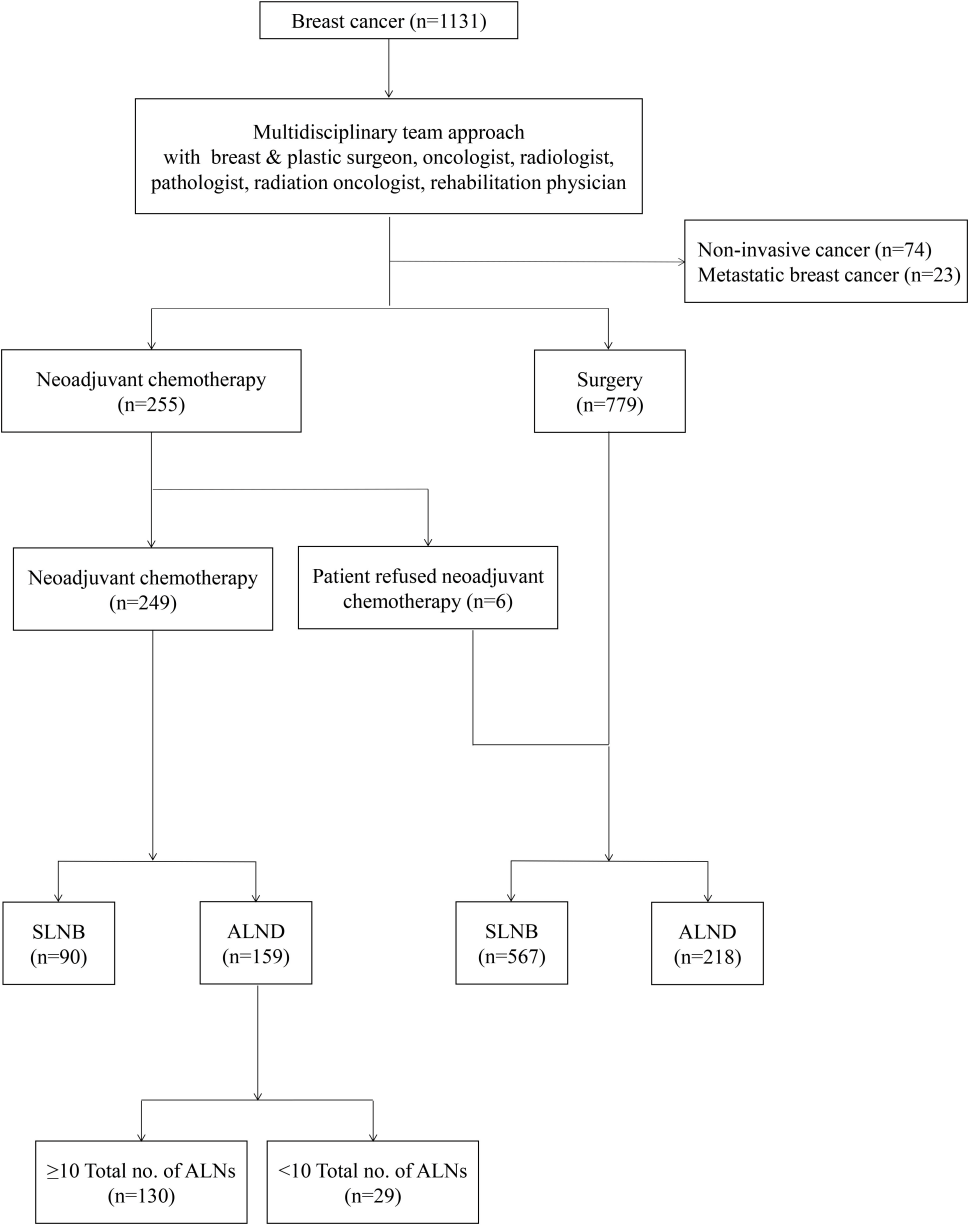
A flow chart showing management of breast cancer with a multidisciplinary team approach. SLNB, Sentinel lymph nodes biopsy; ALND, axillary lymph nodes dissection.

Between 2010 and 2016, the data of 159 patients, with locally advanced breast cancer who underwent breast surgery and ALND after NAC, were selected from 1,131 patients with breast cancer, who were diagnosed and treated at the Kyungpook National University Hospital. The data included the patients' characteristics, medical history, follow-up oncological results, and histopathological characteristics, including molecular subtypes. Before the initiation of treatment, all the patients had undergone core needle biopsy for the diagnosis of breast cancer and fine-needle aspiration cytology for that of ALN. The patients who had noninvasive breast cancer or *de novo* metastatic breast cancer were excluded (Figure 1).

All procedures in this study that involved human participants were performed in accordance with the ethical standards of the Institutional Review Board of the Kyungpook National University Chilgok Hospital (KNUCH 2015-05-205). The experimental protocol was also approved by the Institutional Review Board of the Kyungpook National University Chilgok Hospital, and all the experiments were performed in accordance with relevant guidelines and regulations.

### Treatment and Follow-Up Strategy

All the patients had undergone NAC before the surgery. The NAC regimens were anthracylin + cyclophosphamide (AC) (*n* = 5; 3.1%), AC + taxane (*n* = 116; 73%), AC + taxane + trastuzumab (*n* = 26; 16.4%), and others (*n* = 12; 7.6%). After 3 to 4 cycles of NAC, mammography, breast and neck ultrasound, chest/abdomen computed tomography (CT), and bone scan were rechecked to evaluate the treatment response. Additional cycles were completed if the breast cancer showed partial or complete response. After completion of NAC, breast surgery was performed, including a breast-conserving surgery and mastectomy with ALND (Levels I and II). When the tumor showed stable or progressive disease status during NAC, surgery was considered without completion of NAC. According to the residual tumor burden and molecular subtype in the final pathological report, adjuvant chemotherapy or radiotherapy was offered, and trastuzumab and endocrine therapy were applied, if necessary.

Cancer surveillance was performed in all the patients with blood test monitoring, tumor marker assessment, mammography, breast ultrasound, chest x-rays or CT, abdominal ultrasound or CT, and bone scans biannually for the first 2 years and annually for additional 3 years. The oncological outcomes were assessed based on locoregional recurrence, distant metastasis, or death during the follow-up period.

### Evaluation of Treatment Response and Pathological Results

Treatment response was determined based on the RECIST 1.1 criteria (9). The clinical complete response was defined when there was no evidence of tumor in physical examination with radiological complete response. Clinical partial response was defined when the largest tumor diameter was reduced by more than 30% in radiological images. Clinical stable disease was defined when the largest tumor diameter increased <20%. However, when the largest tumor diameter showed an increase of the largest tumor diameter of 20% or more, it was regarded as a clinically progressive disease.

For each case, all the available hematoxylin and eosin-stained specimens, including frozen-diagnosed and subsequent frozen-permanent samples, were retrospectively reviewed by two pathologists (NJP and JYP) with 10 and 18 years of experience in breast pathology, respectively, in a blinded manner without information about the clinicopathological data or outcomes. The histopathological reviews were conducted independently. Cases with a discrepancy were repeatedly reviewed until a consensus was reached.

The histopathological regression of primary tumor was assessed according to the Miller–Payne grading scales based on the overall cellularity in the excision and mastectomy samples compared with the pretreatment biopsy (10). Grade 1 indicated no change or some alteration to individual malignant cells, but no reduction in overall cellularity (pathologic non-response, pNR). Grade 2 indicated a minor loss of tumor cells, but overall high cellularity, with 30% loss (pPR). Grade 3 indicated an estimated reduction in tumor cells (pathologic partial response, pPR) between 30 and 90%. Grade indicated a marked disappearance of tumor cells such that only small clusters or widely dispersed individual cells remain, with more than 90% loss of tumor cells (almost pCR). Grade 5 indicated that no malignant cells were identifiable in sections from the site of the tumor, with only vascular fibroelastotic stroma remaining, often containing macrophages, but ductal carcinoma *in situ* may be present (pCR).

The evaluation of treatment response in ALNs was more complicated because each specimen had a variable number of lymph nodes, and each lymph node had different treatment regression and therapy-related changes. The presence of nodal tissue was assessed for each specimen, and then the residual tumor burden and any therapy-related histopathological findings at the individual lymph node were independently evaluated. The regression parameters in lymph nodes included size and overall cellularity (percent scale) of residual tumor cells, presence of extranodal tumor extension, intranodal lymphovascular invasion, fibrosis, necrosis, foamy histocytic aggregates, microcalcification, and fibroelastic vascular change (11, 12).

### Statistical Analysis

All statistical analyses were performed using SPSS (version 25.0; SPSS, Chicago, IL). Categorical variables were analyzed using the chi-squared test in univariate analysis, and oncological outcomes were assessed using Kaplan–Meier analysis to identify factors affecting locoregional recurrence, distant metastasis, or death. A *P* < 0.05 was considered statistically significant.

## Results

The mean age of 159 patients was 48. years (SD, ± 9.6 years), and 67 patients (42.1%) were in a postmenopausal state. The patients underwent ALND with mastectomy (*n* = 138; 86.8%) and breast-conserving surgery (*n* = 21; 13.2%). Immediate or delayed breast reconstruction was performed in 26 patients (16.4%). The mean hospital stay after surgery was 12.8 days (SD, ± 3.4 days). Although the mean clinical tumor size was 5.1 cm (SD, ± 2.6 cm) in mammography, breast ultrasound, and breast magnetic resonance, the pathological tumor size after NAC was 2.9 cm (SD, ± 2.2 cm). After the surgery, the patients received additional adjuvant treatments according to the residual tumor burden and immunohistochemistry results [chemotherapy, *n* = 21 (13.2%); target therapy, *n* = 40 (25.2%); radiotherapy, *n* = 130 (81.8%); and hormone treatment, *n* = 119 (74.8%)].

There were 130 cases that showed at least 10 ALNs after NAC in a dissected specimen and 29 cases that showed <10 ALNs. However, the clinical and pathological characteristics were not different between the 2 groups. There were 33 cases (20.8%) of pCR in the breast and 38 cases (23.9%) of pCR in the axilla after NAC. Although there was no significant difference between the 2 groups in the clinical T stage (*P* = 0.590) and the pathological T stage (*P* = 0.183), the pCR in breast lesions showed higher incidence in the group with <10 removed ALNs (18.5 vs. 31%; *P* = 0.009). In addition, the pCR in ALNs showed higher incidence in the group with <10 removed ALNs (21.5 vs. 34.5%; *P* = 0.014), even if there was no significant difference between the two groups in the clinical and pathological N stage (*P* = 0.931 and 0.513). Furthermore, the pCR in both breast and ALNs was higher in the group with <10 removed ALNs (*P* = 0.001) (Table 1). However, there were no differences between the two groups in the subtypes of breast cancer, which showed a nodal pCR (Table 2).

**Table 1:** Clinicopathological characteristics of patients with breast cancer who received axillary lymph node dissection followed by neoadjuvant chemotherapy.

**Variables**	**Total (*n =* 159)**	≥59 ALNs^*^(*n =* 130)	**<10 ALNs (*n =* 29)**	***p-*value**
Mean age (years, ±SD)	48.0 ± 9.6	47.2 ± 9.6	51.7 ± 8.9	0.617
Postmenopausal state (*n*, %)	67 (42.1)	51 (39.2)	16 (55.1)	0.378
Type of breast surgery (*n*, %)				0.104
Breast conserving surgery	21 (13.2)	16 (12.3)	5 (17.2)	
Mastectomy	138 (86.8)	114 (87.7)	24 (82.8)	
Breast reconstruction (*n*, %)	26 (16.4)	23 (17.7)	3 (10.3)	0.501
Mean period of hospital stay (day, ±SD)	12.8 ± 3.4	12.6 ± 3.3	13.3 ± 3.7	0.675
Mean clinical tumor size (cm, ±SD)	5.1 ± 2.6	5.2 ± 2.6	4.7 ± 2.3	0.606
Clinical T stage (*n*, %)				0.590
T1	19 (12.0)	12 (9.2)	7 (24.1)	
T2	90 (56.6)	77 (59.2)	13 (44.8)	
T3	35 (22.0)	28 (21.5)	7 (24.1)	
T4	15 (9.4)	13 (10.0)	2 (6.9)	
Clinical N stage (*n*, %)				0.931
N0	6 (3.8)	5 (3.9)	1 (3.5)	
N1	58 (36.5)	49 (37.7)	9 (31.0)	
N2	59 (37.1)	49 (37.7)	10 (34.5)	
N3	36 (22.6)	27 (20.8)	9 (31.0)	
Clinical stage (*n*, %)				0.084
IIA	9 (5.7)	7 (5.4)	2 (6.9)	
IIB	36 (22.6)	30 (23.1)	6 (20.7)	
IIIA	67 (42.1)	55 (42.3)	12 (41.4)	
IIIB	11 (6.9)	11 (8.5)	-	
IIIC	36 (22.6)	27 (20.8)	9 (31.0)	
Regimen of NAC (*n*, %)				0.612
Anthracycline + Cyclophosphamide (AC)	5 (3.1)	5 (3.9)	-	
AC + Taxane	116 (73.0)	94 (72.3)	22 (75.9)	
AC + Taxane + Trastuzumab	26 (16.4)	23 (17.7)	3 (10.3)	
Others	12 (7.6)	8 (6.2)	4 (13.8)	
Adjuvant chemotherapy (*n*, %)	21 (13.2)	15 (11.5)	6 (20.7)	0.075
Adjuvant target therapy (*n*, %)	40 (25.2)	32 (24.6)	8 (27.6)	0.176
Adjuvant radiotherapy (*n*, %)	130 (81.8)	108 (83.1)	22 (75.9)	0.093
Adjuvant hormone treatment (*n*, %)	119 (74.8)	99 (76.2)	20 (69.0)	0.146

* *Axillary lymph nodes*.

*^†^Neoadjuvant chemotherapy. Thirty-three cases of pathological complete response on breast were excluded in this group*.

**Table 2:** Pathologic results of patients with breast cancer who received axillary lymph nodes dissection followed by neoadjuvant chemotherapy.

**Variables**	**Total (*n* = 159)**	**≥ = ALNs (*n* = 130)**	** <10 ALNs (*n* = 29)**	***p-*value**
Mean pathologic tumor size (cm, ±SD)	2.9 ± 2.2	2.9 ± 1.6	3.0 ± 2.6	0.418
Pathologic T stage (*n*, %)				0.183
pCR (including DCIS only)	33 (20.8)	24 (18.5)	9 (31.0)	0.009
T1	50 (31.5)	43 (33.1)	7 (24.1)	
T2	57 (35.9)	48 (36.9)	9 (31.0)	
T3	49 (30.8)	45 (34.6)	4 (13.8)	
Pathologic N stage^*^ (*n*, %)				0.513
pCR in axilla	38 (24.8)	28 (22.4)	10 (35.7)	0.014
N1	68 (44.4)	58 (46.4)	10 (35.7)	
N2	39 (25.5)	30 (24.0)	9 (32.1)	
N3	14 (9.2)	14 (11.2)	-	
Pathological stage (*n*, %)				0.437
pCR (both breast and axilla)	21 (13.2)	14 (10.8)	7 (24.1)	0.001
IA	13 (8.2)	11 (8.5)	2 (6.9)	
IIA	35 (22.0)	30 (23.1)	5 (17.2)	
IIB	29 (18.2)	25 (19.2)	4 (13.8)	
IIIA	46 (28.9)	35 (26.9)	11 (37.9)	
IIIB	1 (0.6)	1 (0.8)	-	
IIIC	13 (8.2)	13 (10.0)	-	
Estrogen receptor, positive (*n*, %)				
In biopsy before NAC	105 (66.0)	88 (67.7)	17 (58.6)	0.909
In surgical specimen after NAC^†^	86 (54.1)	61 (46.9)	13 (44.8)	0.719
Progesterone receptor, positive (*n*, %)				
In biopsy before NAC	100 (62.9)	85 (65.4)	15 (51.7)	0.759
In surgical specimen after NAC^†^	74 (46.5)	64 (49.2)	10 (34.5)	0.954
HER2/neu gene, positive (*n*, %)				
In biopsy before NAC	44 (27.7)	38 (29.2)	10 (34.5)	0.709
In surgical specimen after NAC^†^	36 (22.6)	25 (19.2)	11 (37.9)	0.420
Subtypes of breast cancer with nodal pCR^*^ (*n*, %)	38 (24.8)	28 (22.4)	10 (35.7)	0.501
Luminal A	8 (5.2)	4 (3.2)	4 (14.3)	
Luminal B	13 (8.5)	12 (9.6)	1 (3.6)	
HER2	8 (5.2)	5 (4.0)	3 (10.7)	
Triple negative	9 (5.9)	7 (5.6)	2 (7.1)	

* *Total number of patients with metastatic lymph nodes was 153*.

† *Neoadjuvant chemotherapy. Thirty-three cases of pathological complete response on breast were excluded in this group*.

**Figure 2:**
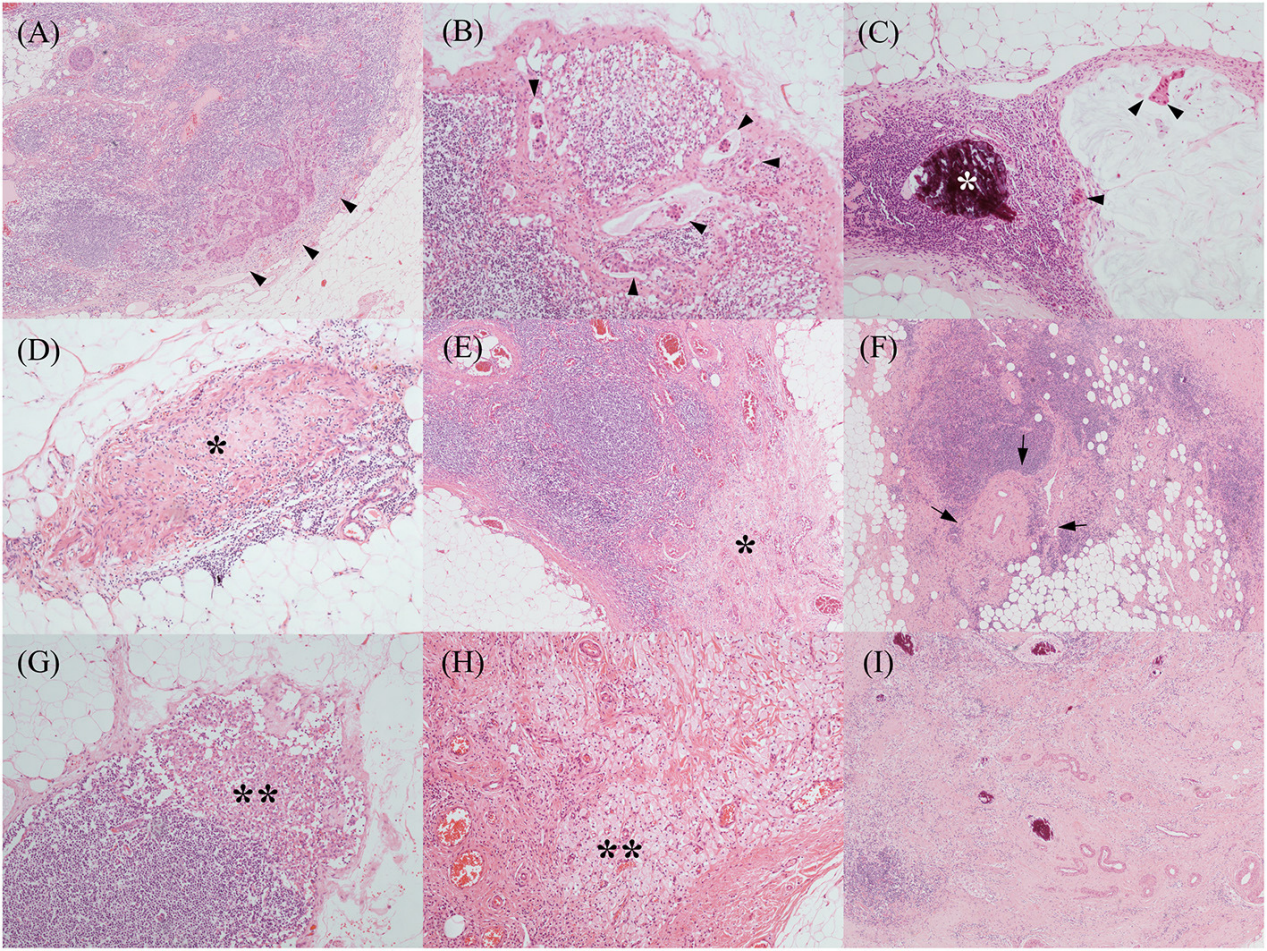
Various treatment-related histopathological findings of axillary lymph nodes. Metastatic tumor cells were often identified at the subcapsular area [**(A)**, black arrowheads], which showed occasional intranodal lymphovascular emboli [**(B)**, black arrowheads]. Another metastatic mucinous carcinoma **(C)** showed floating tumor cell clusters in the mucin pool (black arrowheads) and microcalcification (the white asterisk). Regressed lymph nodes showed a variable degree of fibrosis [**(D,E)**, black asterisks] and fibro-elastic vascular change [**(F)**, black arrows]. Histiocytic infiltrations [**(G,H)**, black double asterisks] were also noted. Above histopathological features, such as fibrosis, vascular change, and microcalcification, were frequently mixed **(I)**. [All, H&E stain; original magnification, **(A,E,F,I)**, x 40; **(B–D,G,H)**, x 100].

The various treatment-related histopathological findings of ALNs are shown in Figure 1. Metastatic tumor cells in ALNs are often identified at the subcapsular area or show intranodal lymphovascular emboli or floating tumor cell clusters (Figures 2A–C). Meanwhile, regressed ALNs usually show fibrosis, fibroelastic vascular change, or histocytic infiltrations (Figures 2D–I).

**Figure 3:**
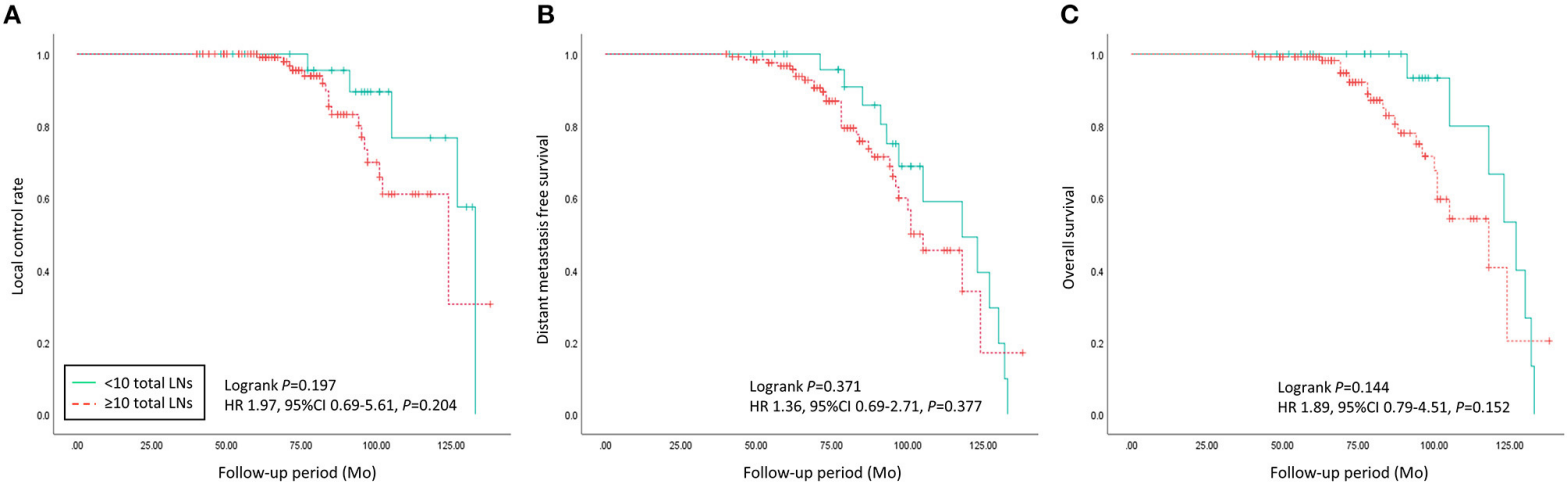
Oncologic outcomes of patients with advanced breast cancer who underwent axillary lymph nodes dissection following neoadjuvant chemotherapy were compared between < 10 and ≥ 10 of the total axillary lymph nodes count group. **(A)** Local control rate, **(B)** distant metastasis, and **(C)** overall survival did not show significant differences between the two groups (*p* = 0.197, 0.371, and 0.144).

During more than 7 years of mean follow-up, there were 22 cases (13.8%) of locoregional recurrence, 45 cases (28.3%) of distant metastasis, and 30 cases (18.9%) of death. There was no significant difference between the two groups (≥ 10 *vs*. < 10 removed ALNs) in locoregional recurrence, distant metastasis, and death (*P* = 0.197, 0.371, and 0.144) (Figure 3, Table 3). Comparing each by the N stage, the pCR in ALNs was highest in the cN2 group (12.3%) of 10 or more removed ALNs and lowest in the cN2 group (6.9%) of <10 removed ALNs (Table 4).

**Table 3:** Oncological outcomes of patients with breast cancer who received axillary lymph nodes dissection followed by neoadjuvant chemotherapy.

**Variables**	**Total (*n* = 159)**	**≥ot ALNs (*n* = 130)**	**<10 ALNs (*n* = 29)**	***p-*value**
Mean follow-up period (months, ±SD)	88.8 ± 21.5	86.2 ± 19.6	90.2 ± 26.1	0.054
Locoregional recurrence (*n*, %)	22 (13.8)	17 (13.1)	5 (17.2)	0.197
Ipsilateral breast	8 (5.0)	5 (3.9)	3 (10.3)	
Ipsilateral axillary lymph node	7 (4.4)	5 (3.9)	2 (6.9)	
Ipsilateral supraclavicular lymph node	12 (7.6)	9 (6.9)	3 (10.3)	
Distant metastasis (*n*, %)	45 (28.3)	32 (24.6)	13 (44.8)	0.371
Lung	23 (14.5)	16 (12.3)	7 (24.1)	
Liver	16 (10.1)	11 (8.5)	5 (17.2)	
Bone	17 (10.7)	12 (9.2)	5 (17.2)	
Brain	2 (1.3)	2 (1.5)	-	
Mediastinal lymph nodes	5 (3.1)	3 (2.3)	1 (3.5)	
Others	7 (4.4)	4 (3.1)	4 (13.8)	
Death (*n*, %)	30 (18.9)	22 (16.9)	8 (27.6)	0.144

**Table 4:** Changes of nodal stages in patients with breast cancer who underwent axillary lymph node dissection followed by neoadjuvant chemotherapy.

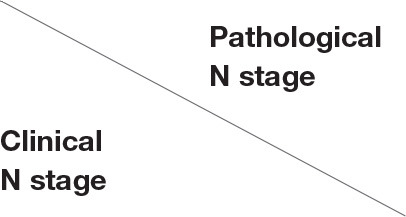	**≥10 ALNs (*****n*** **=** **130)**				** <10 ALNs (*****n*** **=** **29)**			
	**pCR**	**N1**	**N2**	**N3**	**pCR**	**N1**	**N2**	**N3**
N0	-	3 (2.3)	1 (0.8)	1 (0.8)	-	1 (3.5)	-	-
N1	2 (1.5)	35 (26.9)	12 (9.2)	-	4 (13.8)	2 (6.9)	3 (10.3)	-
N2	16 (12.3)	17 (13.1)	12 (9.2)	4 (3.1)	2 (6.9)	5 (17.2)	3 (10.3)	-
N3	10 (7.7)	3 (2.3)	5 (3.9)	9 (6.9)	4 (13.8)	2 (6.9)	3 (10.3)	-

*pCR, pathological complete response*.

## Discussion

The metastatic status of ALNs is an important prognostic factor in breast cancer, and complete removal of metastatic ALNs improves oncological outcomes (13). In particular, when the metastatic ALN is identified at Level II or III, NAC is initiated before surgery because of the difficulty of complete resection of metastatic ALNs. The main role of NAC is reducing the tumor burden, which can lead to increasing the rate of breast-conserving surgery through tumor reduction. Furthermore, the physician can evaluate the treatment response of tumors with NAC (14–16). However, because the tumor status or characteristics can be changed by therapeutic effect, it is very important to investigate the initial tumor stage and characteristics before initiation of treatment.

After the results of the Z0011 trial were published, the feasibility of sentinel lymph node biopsy was established with short- and long-term results (17, 18). However, it may be difficult to confirm the standard additional surgical intervention when the metastasis of the sentinel lymph node is confirmed in pathology because of uncontrolled conditions of the population in the Z0011 trial. The ALN dissection is still a standard treatment in metastatic ALNs of breast cancer, even if the Z0011 trial had been reported. According to the AJCC staging system, at least 10 ALNs should be removed and evaluated to accurately determine the nodal stages (7, 8).

Although the AJCC staging system recommended evaluating more than 10 ALNs when staging nodal status, the total number of ALNs is occasionally <10 in breast cancer treated with NAC, even if complete ALND was performed. Many researchers have reported that the total lymph node count decreases even after NAC in breast cancer (19–22). As subsequent research of those results, the authors investigated the oncological outcomes in breast cancer for which it was reported that the total number of ALNs was <10 after ALND following NAC. The hypothesis of this study was that, if normal lymph nodes were regressed by chemotherapy, the tumor cells in the lymph nodes would have been more affected, which leads to a higher pCR rate in lymph nodes and better oncological outcomes. Although the incidence of pCR in ALNs was higher in breast cancer with metastatic ALNS that showed <10 total ALNs, the oncological outcomes did not show significant differences between the groups with <10 *vs*. 10 or more. This discrepancy may be due to the difference in sample size between the two groups, which is one of the limitations of this study. However, if the sample size increases in further study, we may get more consistent results.

The histopathological findings of ALNs after NAC vary, including various degrees of fibrosis, fibroelastic vascular change, histiocytic infiltrations, or mixture type with microcalcifications. Because the treatment-related histopathological findings have extreme variations, well-experienced pathologists should review and conclude to predict the disease prognosis and to establish the additional treatment strategy.

The extent of ALND may differ according to the surgeon from Levels I and II to Levels I-III. Although there is no specific definition of ALND, thus far, the thoracodorsal vessels and the nerve bundle, the long thoracic nerve should be well exposed after completion of ALND. However, surgeons sometimes think about whether they have performed an incomplete surgery when the total ALN count is <10, even if the complete ALND was performed. According to this study, if the ALND is completely performed after ALND following NAC in locally advanced breast cancer, the oncological outcomes were not inferior to those of the group with 10 or more ALNs. The pCR rates of both ALNs and breast were significantly higher in the group with <10 ALNs, which are expected to have better oncological outcomes in longer follow-up periods. However, in this study, there was no significant difference in oncologic outcomes between the two groups, and this may be due to the small population.

The limitation of this study is that the population of the group with <10 ALNs was small compared to the group with 10 or more ALNs. However, even if the total number of ALNs was <10, the results of this study indicate that additional surgery is not required, and that the surgeon does not need to feel guilty if extensive surgery was performed conscientiously.

## Conclusions

Our study provided a novel finding that the oncological outcomes of the group with <10 ALNs maybe not be inferior to the group with 10 or more ALNs, including locoregional recurrence, distant metastasis, and overall survival. The pCR rate in breast and ALNs was higher in the group with <10 ALNs compared with that of 10 or more. Although due to the small sample size, the accurate significant findings could not be obtained; the results suppose that the surgeon does not need to consider it as incomplete surgery, even if the total ALN count was <10 after ALND following NAC.

## Data Availability Statement

The raw data supporting the conclusions of this article will be made available by the authors, without undue reservation.

## Author Contributions

JL: the guarantor of the integrity of the study and data analysis. JL and NP: study concepts. JL, NP, HP, and J-YP: study design. JL, BK and SL: definition of intellectual content and manuscript preparation. JJ and WK: literature research. J-YP, NP, SL, YC, HK, BK and WK: clinical studies. WK, YC, and SL: data acquisition. JJ: manuscript editing. JJ and HP: manuscript review. All authors have read and approved the manuscript.

## Funding

This work was supported by the National Research Foundation of Korea (NRF) grant funded by the Korea government (NRF-2019R1A2C1006264, 2019R1F1A1063853, and 2017M3A9G8083382).

## Conflict of Interest

The authors declare that the research was conducted in the absence of any commercial or financial relationships that could be construed as a potential conflict of interest.

## Publisher's Note

All claims expressed in this article are solely those of the authors and do not necessarily represent those of their affiliated organizations, or those of the publisher, the editors and the reviewers. Any product that may be evaluated in this article, or claim that may be made by its manufacturer, is not guaranteed or endorsed by the publisher.

## Abbreviations

NAC: neoadjuvant chemotherapy
pCR: pathological complete response
AJCC: American Joint Committee on Cancer
ALND: axillary lymph node dissection
pNRL: pathologic non-response
pPR: pathologic partial response.
